# Inverse Design of Multi-Wavelength Achromatic Metalens Integrated On-Chip with Planar Waveguide

**DOI:** 10.3390/nano15171337

**Published:** 2025-08-31

**Authors:** Mikhail Podobrii, Elena Barulina, Aleksandr Barulin

**Affiliations:** Moscow Center for Advanced Studies, Kulakova Str. 20, 123592 Moscow, Russia

**Keywords:** achromatic metalens, photonic integrated circuit, inverse design, guided mode focusing, fluorescence correlation spectroscopy, single-molecule sensing

## Abstract

Waveguide-integrated metasurfaces offer a promising platform for ultracompact on-chip optical systems, enabling applications such as fluorescence sensing, holography, and near-eye displays. In particular, integrated achromatic metalenses that couple guided modes to free-space radiation are highly desirable for single-molecule fluorescence sensing, where high numerical aperture (NA), efficient light focusing, and consistent focal volume overlap across excitation and emission wavelengths are critical. However, designing integrated high-NA metalenses with multi-wavelength operation remains fundamentally challenging due to the wavelength-dependent propagation of guided modes. Here, we present an inverse design framework that simultaneously optimizes the geometries and positions of silicon nitride nanofins atop a slab waveguide to achieve diffraction-limited focusing at three wavelengths with unity NA. The resulting metalens outperforms conventional segmented designs in focusing efficiency and sidelobe suppression, particularly at wavelengths corresponding to the excitation and emission bands of the model fluorophore Alexa Fluor 647. Numerical analysis shows that the design yields a high molecule detection efficiency suitable for epi-fluorescence single-molecule sensing. This work highlights the potential of inverse-designed metalenses as a versatile on-chip platform for advanced applications in fluorescence spectroscopy, augmented reality, or optical trapping.

## 1. Introduction

Optical metasurfaces are flat optical elements composed of subwavelength nanostructures that have emerged as a powerful alternative to traditional bulky lenses, enabling compact, lightweight, and multifunctional optical systems [1]. For instance, metasurfaces enable vortex beam generation with a large damage threshold [2] or tunable Airy beam’s focal spots [3]. Free-space metalenses open unique opportunities for compact optical elements for advanced sensing and imaging techniques [4] such as two-photon fluorescence microscopy [5,6,7], fluorescence correlation spectroscopy (FCS) [8], Raman spectroscopy [9,10], edge detection [11,12], chiral bioimaging [13], photoacoustic [14,15] imaging, phase-contrast microscopy [16], or optical-coherence microscopy [17]. Multilayered metasurfaces provide efficient polarization converters [18] or structured light beams with varying polarization states along the spatial helical transmission trajectory [19] within the THz regime. While free-space metalenses provide an efficient way to miniaturize refractive lenses, the photonic integrated circuits (PICs) offer a compelling route toward fully on-chip sensing systems [20]. Point-of-care diagnostic technologies, including smartphone-based, chip-integrated, or wearable sensors, are gaining attention for health monitoring applications [21] and could benefit from extreme sensitivity to biomarkers or pathogens. More specifically, single-molecule fluorescence analysis enables highly selective and low limit-of-detection sensing of biomolecules of interest, e.g., proteins, DNA, RNA, for diagnostics without the need for amplification [22,23]. Furthermore, the ability to detect fluorescence fluctuations from individual molecular events allows for direct quantification of biomolecule concentrations via FCS [24,25,26]. Altogether, metasurface-based single-molecule sensors integrated with PICs represent a promising technology for next-generation bioanalytical tools with exceptional sensitivity and miniaturization.

Recent advances have demonstrated metasurface integration with planar slab waveguides to outcouple light into the free space with precise wavefront control, paving the way for multifunctional ultracompact devices such as integrated metalenses [27,28,29], metaholograms [30,31], and metagratings [32,33]. Achromatic metagratings integrated with waveguide displays have gained significant interest for their ability to deliver uniform diffraction across RGB wavelengths, a key requirement for high-resolution augmented reality near-eye displays [34,35]. Despite these developments, designing waveguide-integrated metalenses for epi-fluorescence detection remains challenging. Efficient excitation and collection of fluorescence signals require metalenses with high numerical aperture (NA) and achromatic focusing over both excitation and emission bands [8,36,37].

Achromatic free-space metalenses correct for the inherent chromatic dispersion of diffractive structures, which causes light to focus at different locations for distinct wavelengths, operating from the visible to THz regime [8,38,39,40]. However, correcting chromatic aberrations becomes more challenging in on-chip integrated metalenses, due to the wavelength-dependent propagation constant of the guided mode. Traditional design approaches rely on the detour phase [27,41], where identical meta-atoms are placed at specific locations along the waveguide. While simple, these approaches severely limit design flexibility by constraining the available degrees of freedom. More advanced methods leverage variable-size meta-atoms selected from a precomputed library [42,43]. However, for waveguide-integrated metalenses, meta-atoms are typically placed non-periodically along the propagation axis to get additional phase control. This disrupts the local periodicity assumption on which library-based methods rely, leading to inaccurate phase predictions. Moreover, conventional phase-engineering approaches are typically constrained to operate in a regime of weak coupling between the meta-atoms and the waveguide to avoid significant perturbation of the guided mode. This imposes additional limitations on the design space, particularly on the maximum size of the meta-atoms. However, larger meta-atoms can be advantageous in the context of achromatic metalenses, as they offer increased group delay, which is essential for compensating chromatic dispersion. Although a numerical design of a segmented integrated metalens operating at specific wavelength pairs has been shown in the visible [44], such designs suffer from reduced focusing efficiency and limited performance across multiple wavelengths, ultimately diminishing light collection efficiency and, therefore, hindering potential applications in multispectral imaging or single-molecule fluorescence sensing.

In contrast, the inverse design framework offers a fundamentally different approach. Instead of manually engineering phase delays using analytic formulas, inverse design formulates the metalens design as an optimization problem [45,46,47,48]. The structure is treated as a collection of continuously tunable parameters—such as the shapes and positions of dielectric meta-atoms—and is iteratively adjusted to maximize a user-defined objective, such as intensity at a focal point. In particular, inverse design enables the realization of free-space transmissive or reflective metasurfaces with intricate functionalities that are difficult or impossible to achieve using forward phase-matching approaches, which often fail to simultaneously satisfy the required phase profile across the aperture, e.g., large-scale metalenses with a high NA and focusing efficiency [49,50], metasurfaces featuring two-dimensional focusing control [51]. Genetic algorithms assist in finding optimal material and meta-atom period, height, and lateral dimensions to produce broadband geometric-phase-based free-space metalenses or spin-multiplexed structural beam generators [52]. Moreover, the inverse design approach can circumvent the important problem of non-uniform amplitude and its correlation with phase that is typically omitted in the forward design at the cost of reduced focusing efficiency. This is especially important for waveguide-integrated metalenses, where meta-atoms of varying size exhibit different coupling strengths to the guided mode. Prior studies have shown that inverse design can yield high-NA free-space metalenses with improved performance compared to conventional methods [50,53,54].

In this work, we demonstrate the adjoint-based inverse design of a high-numerical-aperture, multi-wavelength achromatic metalens composed of rectangular meta-atoms of a fixed height, integrated on a planar silicon nitride waveguide. Our approach is based on full-wave electromagnetic simulations and does not rely on simplifying assumptions commonly used in unit-cell or library-based designs. This allows the method to fully capture the effects of structural aperiodicity, meta-atom interactions, and guided mode perturbation. The resulting integrated metalens focuses light in a water medium with a unity NA at three distinct wavelengths. The wavelengths 635 nm, 663 nm, and 695 nm have been selected to match a typical excitation wavelength and two wavelengths within the emission band of Alexa Fluor 647, serving as a model fluorophore system for on-chip single-molecule epi-fluorescence detection. The metalens achieves high focusing efficiency of the fundamental TE_0_ guided mode to the free space and near-perfect spatial overlap of the focal volumes across all three wavelengths, which has not been achieved, to the best of our knowledge, by alternative methods [44]. This leads to a significantly enhanced molecule detection efficiency (*MDE*) suitable for single-molecule fluorescence sensing in diffusion mode. Our design uses geometries compatible with state-of-the-art nanofabrication capabilities, highlighting the practical potential of inverse-designed achromatic metalenses for applications including fluorescence sensing, augmented reality, and quantum photonics.

## 2. Materials and Methods

To optimize the geometry of the metalens, we employ an adjoint-based shape optimization framework [53]. The goal is to maximize the optical intensity at a specified focal point by adjusting the sizes and positions of individual dielectric pillars (meta-atoms) that comprise the metalens. This is formulated as an inverse design problem, where the metalens structure, parameterized by a set of design variables w, is iteratively updated to improve a scalar figure of merit (FoM).

### 2.1. Single-Wavelength Inverse Design

In the single-wavelength case, the FoM is defined as the overlap between the output electric field $E^{f}\left(w\right)$ and the desired field $E^{d}$, which corresponds to the time-reversed field emitted by a point dipole placed at the focal point. Mathematically, the FoM is expressed as:

(1) $$I\left(w\right)={\left|F\left(w\right)\right|}^{2},$$

where F is a complex projection:

(2) $$F\left(w\right)=\int_{S}E^{d*}\cdot E^{f}\left(w\right)\;dA,$$

with the integration performed over some plane *S* located above the metasurface.

The key computational challenge is evaluating the gradient ∂F/∂w with respect to each design parameter $w_{i}$. A naive finite-difference approach would require one full-wave electromagnetic simulation per design parameter, which is computationally prohibitive for large-scale designs. Instead, we leverage the adjoint method, enabling us to compute the full gradient with only two simulations: forward and adjoint. The simulations are conducted using the finite-difference time-domain (FDTD) method.

In the forward simulation, the system is excited by a TE_0_ mode of the planar slab waveguide, and the resulting electric field $E^{f}$ is recorded throughout the simulation domain. For the adjoint simulation, a *y*-polarized point dipole source is placed at the focal point, producing the adjoint field $E^{a}$.

In this work, we restrict the design space to non-rotated rectangular meta-atoms. Both the positions and lateral dimensions of individual meta-atoms are allowed to vary independently. This is equivalent to permitting local displacements of the meta-atom boundaries (facets) in their normal directions. For a facet $\Omega_{i}$, the shape derivative of *F* is given by [53]:

(3) $$\frac{\partial F}{\partial w_{i}}=\frac{1}{2}j\omega \left(n_{m}^{2}-n_{c}^{2}\right)\int_{\Omega_{i}}\left(E_{\left|\right|}^{f}\cdot E_{\left|\right|}^{a}+\frac{1}{n_{m}^{2}n_{c}^{2}}D_{⊥}^{f}D_{⊥}^{a}\right)dA,$$

where $w_{i}$ parameterizes the normal displacement of the boundary $\Omega_{i}$ in the direction of the outward normal, $n_{m}$ and $n_{c}$ are the refractive indices of the meta-atom and the surrounding cladding, respectively, $E_{\left|\right|}^{f}$ and $D_{⊥}^{f}$ are the tangential electric and normal displacement fields in the forward simulation, and $E_{\left|\right|}^{a}$ and $D_{⊥}^{a}$ are the corresponding fields from the adjoint simulation, j is the imaginary unit.

To evaluate Equation (3), the electromagnetic fields on the meta-atom boundaries are required, whereas the FDTD solver provides field values only on a discrete simulation grid. Here, we apply a two-sided linear interpolation scheme [53], which estimates the field values on the meta-atom facets using grid values from both sides of the boundary. The surface integrals are then computed using the two-dimensional trapezoidal rule.

Finally, the gradient of the scalar FoM *I* with respect to the design parameters w, is computed using the chain rule:

(4) $$\frac{\partial I}{\partial w}=2Re\left\{F^{*}\frac{\partial F}{\partial w}\right\}$$

### 2.2. Multi-Wavelength Inverse Design

To design achromatic metalenses, we aim to simultaneously maximize the optical intensity at the focal point for a set of target wavelengths $\left\{\lambda_{1},\lambda_{2},\ldots ,\lambda_{n}\right\}$. We perform electromagnetic simulations using a broadband source whose spectrum includes all desired wavelengths. Importantly, it does not significantly increase the total FDTD simulation time compared to single-wavelength runs. The FoM is then evaluated individually for each wavelength according to Equation (1): $I_{\lambda_{1}},\;I_{\lambda_{2}},\ldots ,I_{\lambda_{n}}$. To ensure balanced performance across all wavelengths, the total objective function is defined as the product of the individual FoMs:

(5) $$I_{total}=\prod_{i=1}^{n}I_{\lambda_{i}}$$

The gradient of the total objective with respect to the design variables is computed using the chain rule:

(6) $$\frac{\partial I_{total}}{\partial w}=\left(\prod_{i=1}^{n}I_{\lambda_{i}}\right)\sum_{i=1}^{n}\frac{1}{I_{\lambda_{i}}}\left(\frac{\partial I_{\lambda_{i}}}{\partial w}\right)$$

The optimization process begins with an initial design of the metalens. This can be a simple configuration, such as a uniform array of identical meta-atoms, or a more advanced structure obtained from a forward design method. At each iteration of the optimization loop, both a forward and an adjoint simulation are performed, after which the FoM and its gradient are evaluated according to Equations (5) and (6). The design is then updated using gradient ascent. The update rule for each parameter $w_{i}$ is given by the following:

(7) $$w_{i}^{\left(k+1\right)}=w_{i}^{\left(k\right)}+\alpha^{\left(k\right)}\frac{\partial I_{total}}{\partial w_{i}},$$

where k is the iteration index and $\alpha^{\left(k\right)}$ is the learning rate at the *k*-th iteration.

This process is repeated until convergence is achieved, the improvement in the FoM falls below a specified threshold, or a maximum number of iterations is reached. The result is a locally optimized metalens geometry tailored to focus light at the desired focal point across multiple wavelengths.

For comparison, we also consider two conventional design methods described below.

### 2.3. Monochromatic Design

In the monochromatic design [41], identical meta-atoms are placed such that the accumulated phase along the waveguide matches the target phase profile of an ideal lens at each position. We extract the accumulated waveguide phase by monitoring the transverse electric field phase in a forward simulation of the waveguide without any meta-atoms. The target phase is defined as the phase of the time-reversed field obtained from the adjoint simulation, where a dipole source is placed at the desired focal point.

To perform the placement, the structure is discretized along the *y*-direction with some chosen periodicity *U*. Within each discretized line, meta-atoms are positioned at the intersections between the waveguide-accumulated phase and the target phase curve. To partially account for the perturbation of the waveguide mode caused by the inserted meta-atoms, we perform an iterative refinement: after the initial placement, we re-run the forward simulation including the meta-atoms, recompute the accumulated phase, and update the positions of the meta-atoms by finding the new phase intersections.

### 2.4. Segmented Design

In the segmentation approach [44], the metalens is divided into alternating zones along the *y*-direction, each dedicated to one of the target wavelengths. Within each zone, meta-atoms are arranged using the same phase-matching strategy described for the monochromatic case. This scheme allows each segment to focus light at a different wavelength to the same focal point, albeit at the cost of reduced efficiency due to spatial sharing.

### 2.5. Simulation Details

All simulations are performed using the Lumerical FDTD solver (Ansys, Canonsburg, PA, USA). We employ the solver’s default lowest-accuracy non-uniform mesh, which yields an effective cell size of 27 × 27 × 50 nm in the meta-atom region. This resolution is comparable to the experimentally validated inverse-design approach for high-NA free-space metalenses [53]. For the boundaries, we use the standard stretched-coordinate perfectly matched layer (PML) with 8 layers. To speed up the computations, we further exploit the symmetry of the TE mode by imposing an anti-symmetric boundary condition on the xz-plane, which reduced the simulated volume by a factor of two. For multi-wavelength optimization, the excitation is provided by the solver’s default Gaussian source, with a spectral width approximately 1.5 times larger than the target frequency range. During the optimization, each FDTD simulation was terminated once the autoshutoff criterion of $10^{-3}$ is reached, which is sufficient for stable convergence. For the final design, however, we employ a stricter threshold of $10^{-6}$ to ensure highly accurate evaluation of the focusing performance.

## 3. Results

To demonstrate the capabilities of our method, we design a 20 × 20 μm metalens integrated on a planar silicon nitride (Si_3_N_4_) slab waveguide with a thickness of 100 nm. The low optical losses of Si_3_N_4_ allow operation at nearly any wavelength in the visible. The metalens focuses the fundamental TE_0_ mode of the waveguide into the surrounding water medium with a high numerical aperture of NA = 1. The sizes and NA are chosen to boost the excitation power density and photon collection angle while remaining in the regime of far-field operation (focal distance ~15λ). The structure consists of 600 nm-tall rectangular Si_3_N_4_ meta-atoms, whose positions and dimensions are optimized using the adjoint method. Fabrication feasibility is supported by meta-atom sizes ranging from 80 to 330 nm. The smallest sizes correspond to aspect ratios within the range for high-quality lithography-based nanofabrication capabilities [55], while the largest sizes still satisfy the Nyquist criterion for the metalens of unity NA.

**Figure 1:**
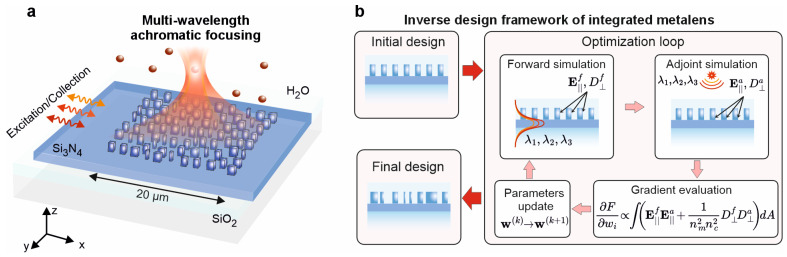
Inverse design of a multi-wavelength achromatic metalens. (**a**) Concept of an achromatic on-chip metalens for single-molecule fluorescence sensing. (**b**) Overview of the inverse design framework, which optimizes the widths and positions of rectangular dielectric meta-atoms to achieve achromatic focusing.

The resulting metalens is achromatic, simultaneously focusing three wavelengths—635 nm, 663 nm, and 695 nm—to the same focal point in all three spatial dimensions. The wavelength 635 nm corresponds to the excitation maximum, while 663 nm and 695 nm lie near the emission maximum of Alexa Fluor 647, a widely used model fluorophore [8]. Achieving high focusing efficiency and spatial overlap at these wavelengths is essential for enabling effective single-molecule epi-fluorescence detection. The conceptual application of the optical device is illustrated in Figure 1a. One operational scenario involves detecting fluorescence fluctuations from single molecules diffusing through the focal volume in a water medium. Therefore, the optimization is performed assuming water immersion above the lens. The meta-atoms are encapsulated in a SiO_2_ (silica-on-glass) cladding of 2 μm thickness. This thickness is chosen to be significantly larger than the evanescent decay length of the near-field at the operating wavelengths (~150 nm), thereby effectively suppressing any direct near-field coupling between fluorophores in the water medium and the nanostructures.

For the optimization process, we employ the inverse design framework described in Section 2 and illustrated schematically in Figure 1b. We investigate three different initialization strategies. The first is a uniform array consisting of identical square meta-atoms, arranged with a period of 350 nm in both directions. The second is a monochromatic design optimized for λ = 663 nm. The third is a segmented design for the desired wavelengths. In all three cases, the meta-atoms are kept identical with a fixed size of 180 × 180 nm, and the *y*-period of the structure is 350 nm.

For the gradient ascent procedure, we employ gradient normalization. At each iteration *k*, the learning rate in Equation (7) is defined as $\alpha^{\left(k\right)}=\beta^{\left(k\right)}/mean\left(\left|\frac{\partial I_{total}}{\partial w}\right|\right)$, which ensures that the average absolute change in the design parameters per iteration equals $\beta^{\left(k\right)}$. The initial step size is set to $\beta^{\left(0\right)}=2\;nm$. If the objective function decreases after a given iteration, β is reduced by half; otherwise, it remains unchanged. The optimization is terminated after 40 iterations. At this point, the increment in focusing efficiency becomes negligibly small (~0.01%).

Since gradient ascent inherently converges to a local maximum, different initial designs lead to distinct final structures. Among the three tested starting points, we selected the segmented design for further analysis: the uniform array exhibited lower overall focusing efficiency, while the monochromatic design showed an imbalance in focusing efficiency, with the highest efficiency occurring at the central wavelength.

**Figure 2:**
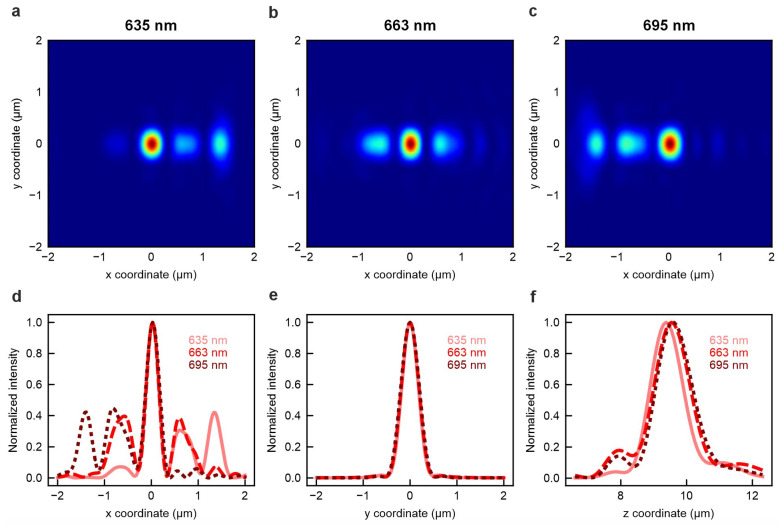
Optical performance of the inverse-designed achromatic on-chip metalens. Lateral point spread functions (PSFs) at wavelengths of (**a**) 635 nm, (**b**) 663 nm, and (**c**) 695 nm. Corresponding one-dimensional PSF intensity profiles along the (**d**) X, (**e**) Y, and (**f**) Z direction.

The initial and final optimized meta-atom arrangements and the convergence plots are presented in Figure S1. For the selected design, we extract the point spread function (PSF) at the target wavelengths. Figure 2a–c present the lateral two-dimensional PSFs at the three operational wavelengths. The full width at half maximum (FWHM) of the focal spot is estimated to be 290 nm in the *x*-direction (Figure 2d) and 420 nm in the *y*-direction (Figure 2e), values that are close to the theoretical diffraction limit. The noticeable astigmatism can be attributed to the intrinsic asymmetry between the *x*- and *y*-directions in the waveguide system, which leads to different focusing behavior along these axes. According to the lateral and axial intensity profiles of the focal volume (Figure 2d–f), the metalens achieves excellent spatial overlap of the focal spots across all three wavelengths. A Strehl ratio amounts to 0.57 for the X-direction focusing profile and 0.78 for the Y-direction focusing profile, respectively. The initial and final refined meta-atom arrangements are schematically represented in Figure S1.

**Figure 3:**
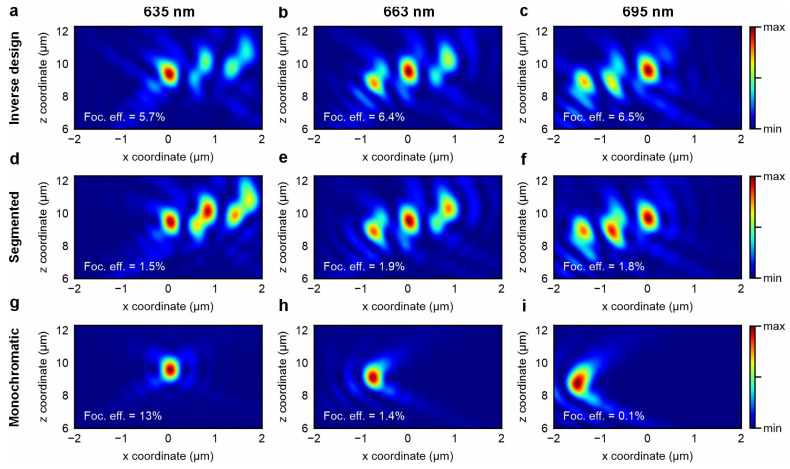
Comparison of point spread functions for three metalens designs at target wavelengths of 635 nm, 663 nm, and 695 nm. Axial point spread functions of (**a**–**c**) inverse-designed achromatic metalens, (**d**–**f**) segmented design, and (**g**–**i**) monochromatic design optimized for 635 nm.

For comparison, we also compute the PSF of the initial segmented design and the monochromatic design tailored for the excitation wavelength of 635 nm. The monochromatic metalens design showcases the problem of the focal spot shift in both X- and Z- directions. In contrast, segmented metalens, to the best of our knowledge, is the only reported approach to achieve achromatic high-NA focusing of a fundamental guided mode from a slab waveguide to free space [44]. To estimate focusing efficiency, we measure the fraction of optical power transmitted through a 1 μm × 1 μm window centered at the focal spot. The selected window size does not exceed 4 FWHM of the lateral spot sizes and does not include the contribution of sidelobes, ensuring an accurate assessment of the power fraction in the main focal spot. The axial PSFs are shown in Figure 3.

The inverse-designed metalens has well-overlapped focal spots and a focusing efficiency of approximately 6% across all three wavelengths (Figure 3a–c), which is a competitive value for integrated metalenses [43]. The segmented design maintains good focal spot overlap but has significantly lower focusing efficiency and stronger unwanted sidelobes compared to the inverse-designed structure (Figure 3d–f). In contrast, the monochromatic design suffers from a substantial focal shift with wavelength (Figure 3g–i), making it unsuitable for applications like epi-fluorescence sensing or broadband imaging. These results highlight the advantage of inverse design, which simultaneously optimizes both the sizes and positions of meta-atoms, in contrast to approaches where meta-atoms are identical. Additionally, considering independent integrated metalens structures that outcouple guided fundamental modes from planar waveguides to free space, our metalens provides the first multi-wavelength operation and competitive focusing efficiency compared to single-wavelength designs (Table S1).

Focusing efficiency, collection efficiency within the fluorescence emission band, and the spatial overlap between excitation and collection volumes are the key factors that determine the molecular detection efficiency (*MDE*) of a lens system [36]. For a specific fluorophore, the *MDE* can be expressed as: $MDE\left(x,y,z\right)=PSF(x,y,z,\lambda_{exc})\cdot CEF(x,y,z)$, where $PSF(x,y,z,\lambda_{exc})$ is the point spread function at the excitation wavelength $\lambda_{exc}$ and CEF(x,y,z) is the collection efficiency for the fluorescent molecule emission [36,44]. In this study, we use Alexa Fluor 647 as a model fluorophore to evaluate the performance of the inverse-designed metalens for in vitro molecular dynamics measurements using fluorescence correlation spectroscopy.

**Figure 4:**
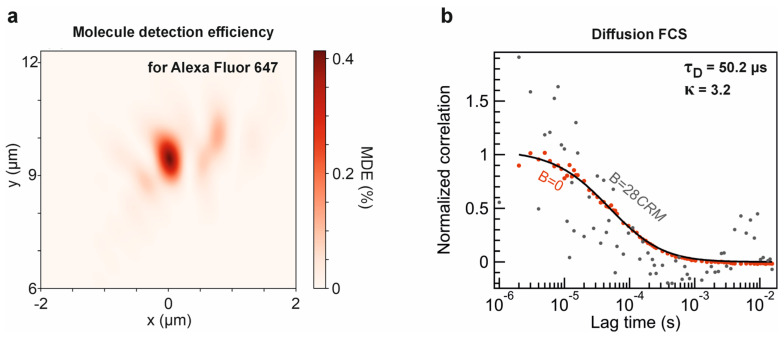
Simulation of single-molecule detectability using the inverse-designed metalens: (**a**) Molecule detection efficiency computed for epi-fluorescence detection with the emission characteristics of Alexa Fluor 647. (**b**) Simulated autocorrelation function of fluorescence photon flow emitted from the metalens detection volume during model single molecule diffusion events without background contribution and with the maximal tolerable background noise intensity (FCS SNR = 3 near G (0)). The background noise intensity of 28 *CRM* corresponds to an SNR of 3 for the near-zero lag time correlation function.

We compute *MDE* using the results of both forward and adjoint simulations, as detailed in Appendix A. Physically, *MDE* quantifies the probability of collecting a fluorescence photon into the propagating TE_0_ mode, assuming the probability distribution of emission follows the excitation PSF with unit amplitude at the center. The collected signal is supposed to be subsequently detected outside the photonic integrated circuit. The inverse-designed metalens achieves a maximum *MDE* exceeding 0.4% (Figure 4a), significantly outperforming conventional forward-designed metalenses reported in the literature [41,44].

The total number of photons collected from a single molecule can be determined as [56]: $CRM=MDE\cdot \psi \cdot \bar{S_{1}}\cdot \tau^{-1}$, where ψ is the quantum yield of the fluorophore, $\bar{S_{1}}$ is the steady state population of the first excited state, and τ is the lifetime of the fluorophore. We simulate the FCS data (autocorrelation functions) of diffusing Alexa Fluor 647 molecules in the aqueous solution (diffusion coefficient 330 μm^2^/s). The simulations are conducted via a Monte Carlo approach (SimFCS3 https://www.lfd.uci.edu/globals/, accessed on 14 July 2025). Similarly to isotropic 3D Brownian motion, molecules were allowed to stochastically move through a 3D voxel grid with 50 nm spacing within a simulation volume of 262 μm^3^. Photon emission events were sampled according to the *MDE* distribution, and simulated photon timestamps over 30 s were used to generate the fluorescence autocorrelation curve (Figure 4b). It is worth noting that the autocorrelation function exhibits a signal-to-noise ratio above 3 at near-zero lag times, acceptable for FCS running [57], even with background noise intensity of 28·*CRM*. The autocorrelation data exhibit characteristic decay due to diffusion and are well-fitted by the standard 3D Brownian diffusion model:

(8) $$G_{D}\left(\tau \right)=\frac{1}{N}\frac{1}{1+\tau /\tau_{D}}{\left(1+\frac{\tau \omega_{xy}^{2}}{\tau_{D}\omega_{z}^{2}}\right)}^{-0.5},$$

where N is the average number of molecules in the detection volume, $\tau_{D}$ is the diffusion time, and $\omega_{xy}$, $\omega_{z}$ are the lateral and axial $1/e^{2}$ radii of the effective *MDE* volume, respectively.

These results demonstrate that the inverse-designed metalens enables efficient epi-fluorescence detection under a PIC-integrated configuration, with sufficient photon collection for FCS measurements with sub-millisecond lag times. Notably, the design achieves high focusing efficiency and achromatic performance without relying on a predefined library or unrealistic meta-atom geometry configurations. This highlights the potential of inverse-designed achromatic metalenses for compact and tunable on-chip applications, including single-molecule detection, augmented reality, and broadband optical communication.

## 4. Discussion

The demonstrated inverse design approach establishes a powerful and flexible framework for optimizing integrated metalenses, enabling achromatic focusing at multiple wavelengths and arbitrary NAs. Crucially, this method directly optimizes the focusing efficiency across all selected wavelengths without relying on simplifying assumptions about the structure, making it well-suited for realizing advanced devices and complex optical functionalities.

The proposed approach relies on full-wave electromagnetic simulations, which are inherently computationally intensive. However, GPU-accelerated FDTD solvers allow scaling of the optimization framework to apertures up to ~100 µm [58], which is sufficient for most practical applications [43,59,60]. For significantly larger apertures, such as wafer-scale devices, various modified inverse design strategies have been proposed for free-space metalenses [50,61,62]. Adapting these methods to on-chip configurations remains an open challenge and a subject of further research.

Although achieving a globally optimal figure of merit remains challenging, we show that metalenses initially constructed using forward design principles and subsequently refined via inverse design exhibit marked improvements across key performance metrics. These include increased focusing efficiency, enhanced beam overlap, and reduced sidelobes or secondary foci. While initiating the optimization from a forward-designed structure can expedite convergence and improve final performance, the method is robust enough to yield high-quality solutions even when no viable forward design exists.

The high-NA integrated metalens optimized within this framework achieves high molecule detection efficiency for Alexa Fluor 647 single molecules under epi-fluorescence configuration. Furthermore, fluorescence correlation spectroscopy simulations of diffusing Alexa Fluor 647 through the metalens focal volume demonstrate its potential for precise on-chip molecular or nanoparticle size and concentration measurements. Given the low thermo-optic coefficients of the constituent optical device materials, the integrated metalens performance is insensitive to temperature, with a potential for outdoor applications (Figure S2). Importantly, the inverse design can be tailored to arbitrary wavelengths, dictated by the target fluorophore’s emission or the specific application. For instance, the inverse-designed integrated metalens yields well-defined achromatic focusing in the green spectral range that could be suitable to collect epi-fluorescence from Alexa Fluor 555 or other green fluorophore analogues (Figure S3). This underscores the potential of the design methodology for future ultra-miniaturized biomarker sensing platforms for precision medicine. Lastly, beyond single-molecule fluorescence sensing, the inverse design approach can be used for the development of augmented reality integrated platforms [35], on-chip multispectral photoacoustic imaging [63], or on-chip trapped-ion quantum information processing schemes [64].

## Data Availability

Data are contained within the article.

## Supplementary Materials

The supporting information can be downloaded at https://www.mdpi.com/article/10.3390/nano15171337/s1, Figure S1. Inverse-designed metalens structure optimization results: (a) Evolution of the figure of merit during the optimization process. (b) Evolution of the average absolute change in design parameters per iteration. (c) Evolution of focusing efficiencies at 635 nm, 663 nm, and 695 nm. (d) Initial meta-atom arrangement used as the starting point. (e) Final meta-atom arrangement obtained after optimization with the inverse-design framework. Table S1. Comparative analysis of integrated metalens performance for independent designs. Figure S2. Axial point spread functions of the inverse-designed integrated metalens at the target wavelengths of (a) 635 nm, (b) 663 nm, and (c) 695 nm, assuming a refractive index change induced by a 20 °C temperature increase. A 20 °C shift is considered a relatively severe environmental stress for potential outdoor applications. The following thermo-optic coefficients were used to model the heating effect: (i) dn/dT = 2.5 × 10^−5^ for silicon nitride, (ii) dn/dT = −10^−4^ for water, and (iii) dn/dT = 8.5 × 10^−6^ for silica cladding layers. Figure S3. Axial point spread functions of the inverse-designed integrated metalens (NA = 1, size 20 μm) operating in the green spectral range at target wavelengths of (a) 532 nm, (b) 568 nm, and (c) 604 nm. The meta-atom, cladding, and waveguide materials are identical to those in the red-band design. These wavelengths are well-suited for excitation and collection of fluorescence photons from Alexa Fluor 555, a green-emitting fluorophore.

## Appendix A

The collection efficiency $CEF\left(r\right)$ is defined as the fraction of optical power emitted by an isotropic point source located at position $r=\left(x,y,z\right)$, that is coupled into the backward-propagating TE_0_ waveguide mode within the fluorophore emission band. Since our integrated metalens is designed to collect only *y*-polarized light, the collection efficiency is equal to one-third of that of a *y*-polarized dipole. Thus, the *CEF* can be expressed as

(A1) $$CEF\left(r_{d}\right)=1/3\int T\left(r_{d},\lambda \right)S\left(\lambda \right)d\lambda ,$$

where $S\left(\lambda \right)$ is the fluorophore emission spectrum, and $T\left(r_{d},\lambda \right)$ is the optical power transmitted from the dipole at point $r_{d}$ into the TE_0_ mode. For the lossless waveguide, the transmission coefficient $T\left(r_{d},\lambda \right)$ can be evaluated as $T\left(r_{d},\lambda \right)={\left|b\right|}^{2}$, where b is a projection of the dipole-emitted field onto the mode [65]

(A2) $$b=\frac{1}{4N}\int dS{(E}_{dip}\times H_{m}-E_{m}\times H_{dip}).$$

Here $E_{m},\;H_{m}$ are the modal fields (with real-valued transverse components), $E_{dip},H_{dip}$ are the fields produced by the dipole, $N=1/2\int dS(E_{m}\times H_{m}^{*})$ is the normalization constant. The integration is performed over the waveguide input cross-section (*yz* plane). However, directly computing $CEF\left(r_{d}\right)$ would require a separate dipole simulation for every point $r_{d}$, which is computationally expensive. To overcome this, we show that the full $CEF\left(r\right)$ map can be computed using only two simulations—the same forward and adjoint simulations employed for the inverse design optimization. Let $J_{m}=\left[n\times H_{m}\right]$ and $M_{m}=-\left[n\times E_{m}\right]$ be the equivalent electric and magnetic surface currents [66] producing the mode, which are actually the sources in the forward simulation, and produce the field $E^{f}$; $J_{dip}=J_{0}\delta \left(r-r_{d}\right)$ is the dipole current, which produces the fields $E_{dip},H_{dip}$. Applying the generalized reciprocity theorem with magnetic currents [66] yields:

(A3) $$\int dS{(E}_{dip}\;J_{m}-H_{dip}M_{m})=\int d^{3}r\;E^{f}\;J_{dip}.$$

Substituting the currents, we obtain

(A4) $$\int dS{(E}_{dip}\;\left[n\times H_{m}\right]+H_{dip}\left[n\times E_{m}\right])={J_{0}E}^{f}(r_{d})\propto E_{y}^{f}\left(r_{d}\right)$$

Swapping operands in the triple vector products, we found that $b\propto E_{y}^{f}\left(r_{d}\right)$ and therefore $T\left(r_{d},\lambda \right)\propto |{\left.E_{y}^{f}\left(r_{d},\lambda \right)\right|}^{2}$. An adjoint simulation, with a dipole placed at the focal point $r_{0}$, provides $T\left(r_{0},\lambda \right)$. We can then express T at any other point using the field from the forward simulation:

(A5) $$T\left(r_{d},\lambda \right)=T\left(r_{0},\lambda \right)\frac{|{\left.E_{y}^{f}\left(r_{d},\lambda \right)\right|}^{2}}{{\left|E_{y}^{f}\left(r_{0},\lambda \right)\right|}^{2}}$$

Then, *CEF* is obtained using Equation (A1), and the final molecule detection efficiency is obtained by multiplying by the normalized PSF data at the excitation wavelength.
