# Supplementary material for: Inverse Design of Multi-Wavelength Achromatic Metalens Integrated On-Chip with Planar Waveguide
Source: Nanomaterials (Basel). 2025 Aug 31;15(17):1337. doi: 10.3390/nano15171337 (PMC12430403; doi:10.3390/nano15171337)
Supplement: Supplementary file 1 [file nanomaterials-15-01337-s001.zip › nanomaterials-3818804-supplementary.pdf]

Supplementary Materials

Inverse design of multi-wavelength achromatic metalens integrated on-chip with planar waveguide

Mikhail Podobrii[1], Elena Barulina[1], Aleksandr Barulin[1,*]

[1] Moscow Center for Advanced Studies, Kulakova str. 20, Moscow 123592, Russia
* Correspondence: alexbarulin73@gmail.com, A.B.

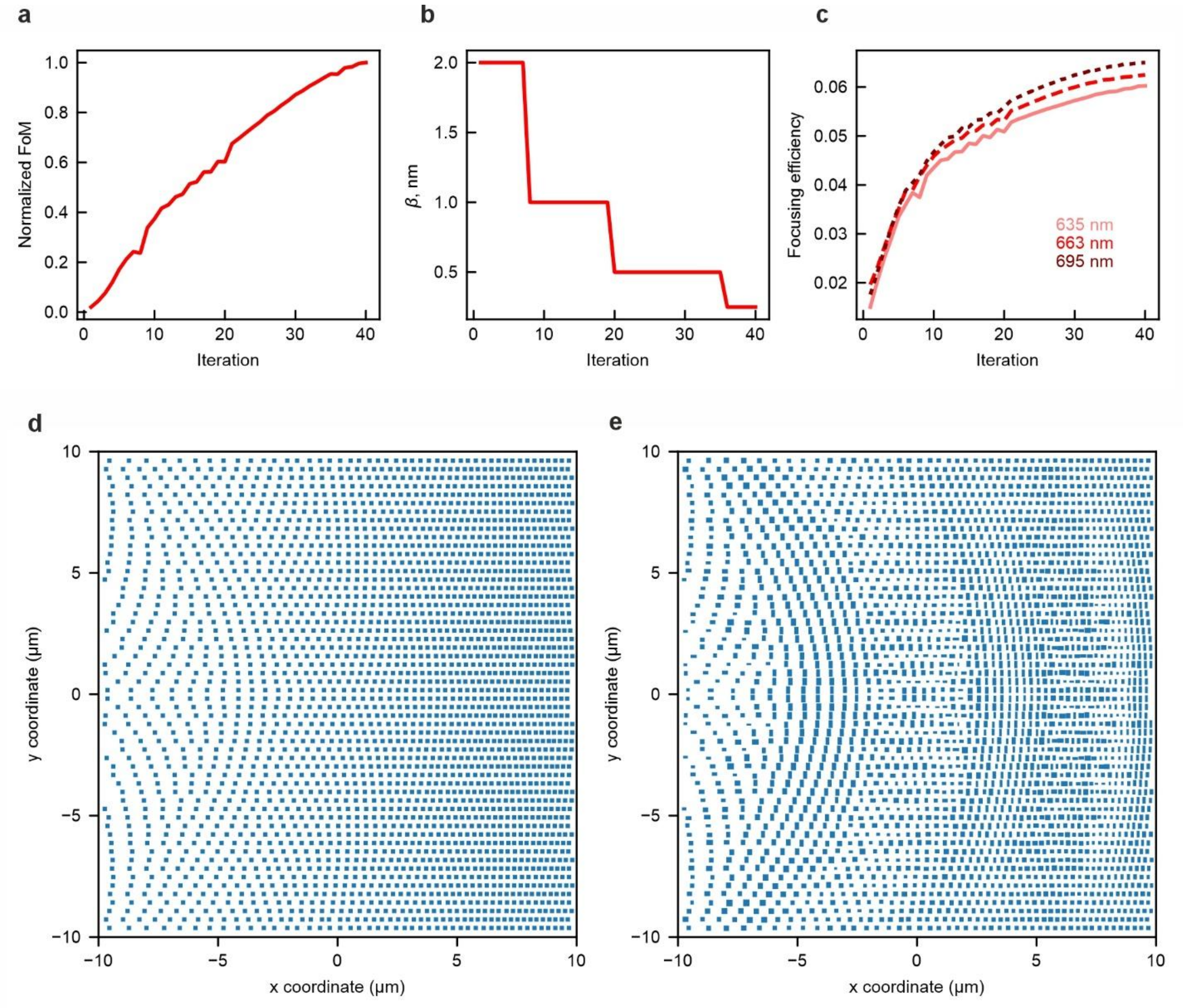

Figure S1. Inverse-designed metalens structure optimization results. (a) Evolution of the figure of merit during the optimization process. (b) Evolution of the average absolute change in design parameters per iteration. (c) Evolution of focusing efficiencies at 635 nm, 663 nm, and 695 nm. (d) Initial meta-atom arrangement used as the starting point. (e) Final meta-atom arrangement obtained after optimization with the inverse-design framework.

Table S1. Comparative analysis of integrated metalens performance for independent designs.

Ref.	Design method	Metalens size	Wavelengths	Chromatic aberration correction	NA	Waveguide/ meta-atom materials	Simulated focusing efficiency
[S1]	Forward design (periodic array)	60 µm x 20 µm	1550 nm	*Not applicable*	~0.3	Si / a-Si	0.14
[S2]	Forward design (aperiodic array)	60 µm x 20 µm	1550 nm	*Not applicable*	~0.3	Si / a-Si	-
[S3]	Forward design (aperiodic array)	15 µm	532 nm	*Not applicable*	~0.7	Si_3N_4 / Si	~0.042
[S4]	Forward design (periodic array)	5 µm	1550 nm	*Not applicable*	~0.45	Si / Au-SiO_2-Au stacked nanobar meta-atoms	-
[S5]	Forward design (periodic array, geometric phase)	8 µm	1550 nm	*Not applicable*	0.625	Lithium niobate / Si	0.016 for 8 µm metalens
[S6]	Forward design (aperiodic array, solid-immersion metalens)	20 µm	500 nm/660 nm; 520nm/570 nm; 650 nm/ 780 nm	Two wavelengths with a separation above 50 nm	1.1	Si_3N_4 / Si_3N_4	~0.08
This work	Inverse design (aperiodic array, solid-immersion metalens)	20 µm	635nm/663 nm/ 695 nm	Three wavelengths	1	Si_3N_4 / Si_3N_4	~0.065

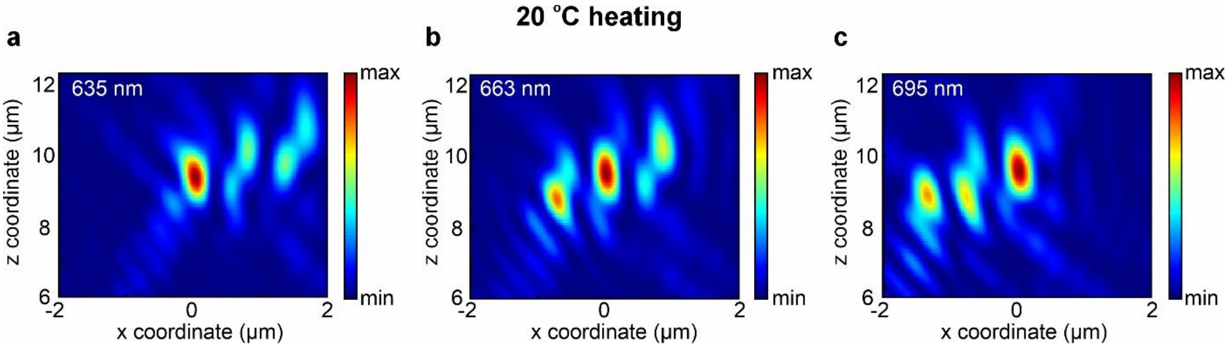

Figure S2. Axial point spread functions of the inverse-designed integrated metalens at the target wavelengths of (a) 635 nm, (b) 663 nm, and (c) 695 nm, assuming a refractive index change induced by a 20 °C temperature increase. A 20 °C shift is considered a relatively severe environmental stress for potential outdoor applications. The following thermo-optic coefficients were used to model the heating effect: (i) $dn/dT=2.5\cdot10^{-5}$ for silicon nitride, (ii) $dn/dT = -10^{-4}$ for water, and (iii) $dn/dT = 8.5\cdot10^{-6}$ for silica cladding layers.

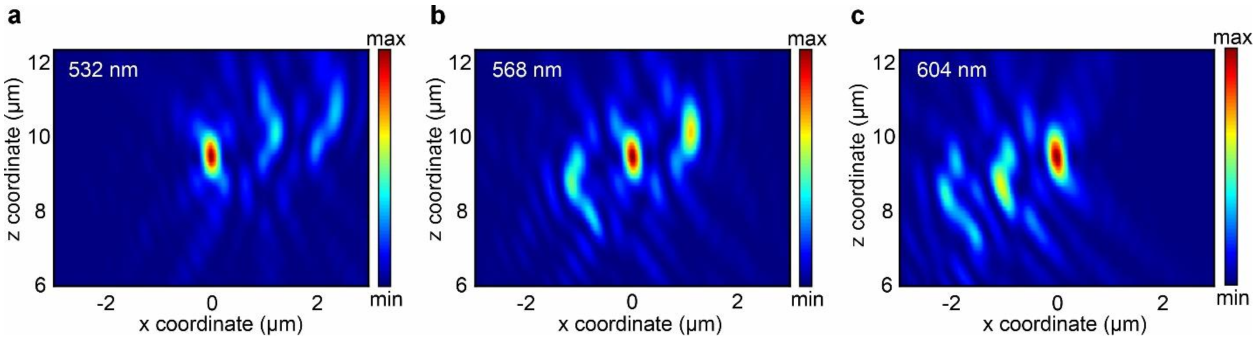

Figure S3. Axial point spread functions of the inverse-designed integrated metalens (NA=1, size 20 μm) operating in the green spectral range at target wavelengths of (a) 532 nm, (b) 568 nm, and (c) 604 nm. The meta-atom, cladding, and waveguide materials are identical to those in the red-band design. These wavelengths are well-suited for excitation and collection of fluorescence photons from Alexa Fluor 555, a green-emitting fluorophore.

Supplementary references:

S1. Hsieh, P.-Y.; Fang, S.-L.; Lin, Y.-S.; Huang, W.-H.; Shieh, J.-M.; Yu, P.; Chang, Y.-C. Integrated Metasurfaces on Silicon Photonics for Emission Shaping and Holographic Projection. *Nanophotonics* **2022**, *11*, 4687–4695, doi:10.1515/nanoph-2022-0344.

S2. Hsieh, P.-Y.; Fang, S.-L.; Lin, Y.-S.; Huang, W.-H.; Shieh, J.-M.; Yu, P.; Chang, Y.-C. Metasurfaces on Silicon Photonic Waveguides for Simultaneous Emission Phase and Amplitude Control. *Optics Express* **2023**, *31*, 12487–12496.

S3. Li, Z.; Liu, Y.; Zhang, C.; Qiao, Y.; Deng, R.; Shi, Y.; Li, Z. On-Chip Direction-Multiplexed Meta-Optics for High-Capacity 3D Holography. *Adv Funct Materials* **2024**, *34*, doi:10.1002/adfm.202312705.

S4. Ding, Y.; Chen, X.; Duan, Y.; Huang, H.; Zhang, L.; Chang, S.; Guo, X.; Ni, X. Metasurface-Dressed Two-Dimensional on-Chip Waveguide for Free-Space Light Field Manipulation. *ACS Photonics* **2022**, *9*, 398–404, doi:10.1021/acsphotonics.1c01577.

S5. Fang, B.; Wang, Z.; Gao, S.; Zhu, S.; Li, T. Manipulating Guided Wave Radiation with Integrated Geometric Metasurface. *Nanophotonics* **2022**, *11*, 1923–1930, doi:10.1515/nanoph-2021-0466.

S6. Barulina, E.; Nguyen, D.D.; Shuklin, F.; Podobrii, M.; Novikov, S.; Chernov, A.; Kim, I.; Barulin, A. Dual-Wavelength On-Chip Integrated Metalens for Epi-Fluorescence Single-Molecule Sensing. *Sensors* **2024**, *24*, 7781.
